# Absorption and birefringence study for reduced optical losses in diamond with high nitrogen-vacancy concentration

**DOI:** 10.1098/rsta.2022.0314

**Published:** 2024-01-22

**Authors:** Tingpeng Luo, Felix A. Hahl, Julia Langer, Volker Cimalla, Lukas Lindner, Xavier Vidal, Marko Haertelt, Remi Blinder, Shinobu Onoda, Takeshi Ohshima, Jan Jeske

**Affiliations:** ^1^ Fraunhofer Institute for Applied Solid State Physics IAF, 79108 Freiburg, Germany; ^2^ Institut für Quantenoptik, University of Ulm, 89081 Ulm, Germany; ^3^ National Institutes for Quantum Science and Technology (QST), 1233 Watanuki, Takasaki, Gunma 370-1292, Japan

**Keywords:** NV centre, optical loss, absorption, birefringence, diamond, quantum sensing

## Abstract

The use of diamond colour centres such as the nitrogen-vacancy (NV) centre is increasingly enabling quantum sensing and computing applications. Novel concepts like cavity coupling and readout, laser-threshold magnetometry and multi-pass geometries allow significantly improved sensitivity and performance via increased signals and strong light fields. Enabling material properties for these techniques and their further improvements are low optical material losses via optical absorption of signal light and low birefringence. Here, we study systematically the behaviour of absorption around 700 nm and birefringence with increasing nitrogen- and NV-doping, as well as their behaviour during NV creation via diamond growth, electron beam irradiation and annealing treatments. Absorption correlates with increased nitrogen doping yet substitutional nitrogen does not seem to be the direct absorber. Birefringence reduces with increasing nitrogen doping. We identify multiple crystal defect concentrations via absorption spectroscopy and their changes during the material processing steps and thus identify potential causes of absorption and birefringence as well as strategies to fabricate chemical vapour deposition diamonds with high NV density yet low absorption and low birefringence.

This article is part of the Theo Murphy meeting issue ‘Diamond for quantum applications’.

## 1. Introduction

Negatively charged nitrogen-vacancy (NV) centres in diamonds have been extensively studied for quantum sensing applications. Since their spin property can be optically read out at room temperature, NV centres have become a leading experimental quantum system and are widely used to detect magnetic field [1–4], electric field [5,6], temperature [7–9], pressure [10] and strain [11,12] and are a promising system to realize advanced sensing concepts such as field fluctuation sensing and identification of quantum coherent systems in the environment [13–15]. NV-ensembles in bulk diamonds can provide significantly stronger signals than individual NV centres for applications that require good sensitivity, which can be further enhanced by the multi-pass readout [16,17], optical cavity coupling [18–21] and laser cavity sensing [20,22–25]. Such techniques can significantly increase the readout signal and/or contrast by improved collection from the NV centres, increased coupling to a collection mode or stimulated emission. For their performance, a material parameter becomes particularly relevant: low optical losses of the signal light in the diamond material. Optical losses in diamonds are typically small and thus play a minor role in normal NV fluorescence collection. For the above-mentioned techniques, however, the level of optical losses can limit or significantly enhance sensitivity advantages. These techniques further benefit from high NV concentrations for a strong signal [26], while the crystal quality also plays an important role as it enables a reduced optical loss in the material.

Diamond absorption of the light signal is the main source that introduces optical loss [25]. Specifically, absorption at around 700 nm is the most relevant regime, as it contains most of the NV fluorescence. As an example, stimulated emission of NV centres [27] and laser-threshold magnetometry (LTM), i.e. the idea to use NV centres as a gain medium [22–25,28,29] are ways to significantly enhance sensitivity. For this, a very low absorption at approximately 700 nm is needed to achieve net optical gain and lasing [25]. Understanding the source of the absorption in this regime plays a crucial role to optimize the material quality thus improving the performance of the sensing method.

Previous studies identified many absorbers in diamond, which can give us hints of sources of the absorption at 700 nm, here we list four point defects as potential candidates: The first one is the H2 centre (${\mathrm{NVN}}^{-}$), which has a zero-phonon-line (ZPL) at 986.3 nm and a very broad absorption side-band from around 600 nm to the ZPL [30]. It often appears when creating NV centres, and it is also formed from the single substitutional nitrogen atoms (denoted as P1 centre, $N_{s}^{0}$ or C-centre). The second one is a band centred at 730 nm, which is always present together with 520, 552 and 840 nm bands, but not correlated perfectly in intensity [30], and its origin has not been well defined. The third candidate is a broad band centred at 710 nm, which may be associated with the vibronic side-band of the nickel-related centre at 794 nm [31]. The last one is then the GR1 band caused by neutral single vacancies (V${\;}^{0}$), which has a ZPL at 741 nm and a broad feature from around 500 to 750 nm [32]. Among these candidates, the first one is commonly observed in chemical vapour deposition (CVD) diamonds, while the second and third appear most likely in high-pressure, high-temperature (HPHT) diamonds. The last one then appears mostly in irradiated diamonds. Apart from the point defect, non-diamond inclusions can also play a role as they contribute to a high absorption in the whole UV–Visible range, also resulting in a greyish coloration of the diamond [33].

Another distinct source of optical loss in diamond is the birefringence. For cavity-based NV-magnetometry, optical modes suffer from birefringence in the material. Moreover, polarization-selective addressing and readout of NV centres are also only possible without birefringence as well as polarization-selective elements, such as Brewster’s angle diamond interfaces and optical elements. Therefore, when pursuing high NV concentrations for improved sensitivity, the influence of the nitrogen content on birefringence also needs to be considered. Diamond is generally considered to be an isotropic crystal without or with weak birefringence. However, extended defects in diamonds such as dislocations and stacking faults generate strain fields that distort the diamond lattice [34,35]. This leads to localized birefringence patterns with randomly varying slow axes [36], which appear inhomogeneously in the crystal. Additionally, applied stress can also make the diamond structure anisotropic thus resulting in birefringence.

Diamonds with high NV concentrations, low absorption and low birefringence lead to a strong signal and reduced optical loss, thus benefiting applications with large sensing volumes. In this work, we investigate the link between nitrogen doping, NV creation and diamond absorption/birefringence, discuss possible causes of absorption and birefringence and suggest potential approaches to reduce them to obtain an optimized diamond.

## 2. Methods

### (a) Sample processing

We have grown over 50 (100) oriented CVD diamonds with varying parameters for both the absorption and birefringence study. The growth of all CVD samples was run in an ellipsoidal-shaped CVD reactor with a 2.45 GHz microwave frequency and equipped with a 6 kW microwave generator [37]. Apart from CVD samples, we also investigated HPHT Ib diamonds (Element Six and Sumitomo) for the birefringence study. All samples are in the form of bulk diamond plates with a geometry of $3\times 3\;{\mathrm{mm}}^{2}$ or $4\times 4\;{\mathrm{mm}}^{2}$, the thickness varied from 200 to 1400 μm. They have been cut from the substrate and polished on both surfaces with a roughness Ra<0.5 nm before the optical measurement. The samples are investigated after growth, and partially after irradiation and annealing. We have conducted electron-beam irradiation with 1 and 2 MeV electron energies and different fluences, the subsequent annealing step was performed at $1000^{\circ }C$ for 2 h.

### (b) Optical measurements

The absorption of the sample was measured with a UV–Vis spectrometer (PerkinElmer Lambda 950) at room temperature. The absorption coefficient was obtained from the transmittance T, which was measured by the spectrometer. For a precise study of absorption at approximately 700 nm, we measured the sample with an integrating sphere at the wavelength of 680–760 nm to gather all transmitted light in this regime. The absorption coefficient $A_{\mathrm{coef}}$ in this case is given by Hahl [38]:

2.1 $$A_{\mathrm{coef}}=-\frac{\log_{10}\left(\sqrt{4T^{2}+{\left(1-2R_{t}+R_{t}^{2}-T^{2}\right)}^{2}}-1+2R_{t}-R_{t}^{2}+T^{2}\right)}{2dT},$$

where d is the sample thickness and $R_{t}\approx 29.13\%$ is the theoretical value of the reflectance based on the Fresnel equation for normal incidence on a diamond with refractive index n=2.4. For both the absorption and birefringence calculation (discussed below), d is given by the average thickness of the diamond plate.

For the spectral study on the other hand, we used the standard detector of the spectrometer without the integrating sphere to measure the transmittance at 200–800 nm (since the functional wavelength of the material in the integrating sphere is only above 400 nm, the sphere is not eligible for the spectral study in a large wavelength range). In this case, the absorption coefficient A is simplified as

2.2 $$A=-\log_{10}(T)/d,$$

where the reflection is not taken into account as the spectral feature is more of interest than the absolute value of absorption.

The birefringence was measured with a polarimeter (Ilis StrainMatic M4/90.50 Zoom) following the Sénarmont method [39]. The phase difference between the two axes with different refractive indices was measured, i.e. the optical retardation Γ. The birefringence Δn is then given by

2.3 $$\Delta n=\frac{\Gamma }{d}.$$

The measurement of the retardation Γ was spatially resolved, which gives a map of birefringence Δn for the sample.

Single substitutional nitrogen P1 centres were measured to investigate the link between nitrogen doping and diamond absorption/birefringence. We used two methods to measure their concentration in this work: electron paramagnetic resonance (EPR) and UV–Vis spectroscopy. The EPR measurement was conducted at room temperature with an EPR spectrometer (Bruker ELEXSYS E580), which is fitted with a Bruker super-high-Q probehead (ER4122 SHQE). The microwave frequency was set to 9.84 GHz, and the P1 concentration was determined using the built-in spin-counting feature, from the acquisition software (xEPR). UV–Vis measurements follow our methods paper [40]. We extracted the absorption band at 270 nm from the other spectral features, determine the absorption coefficient plots via equation (2.2) and the concentration of P1 centres via the absorption cross section determined from multiple reference samples.

## 3. Diamond absorption

### (a) Absorption for varying nitrogen contents

**Figure 1.**
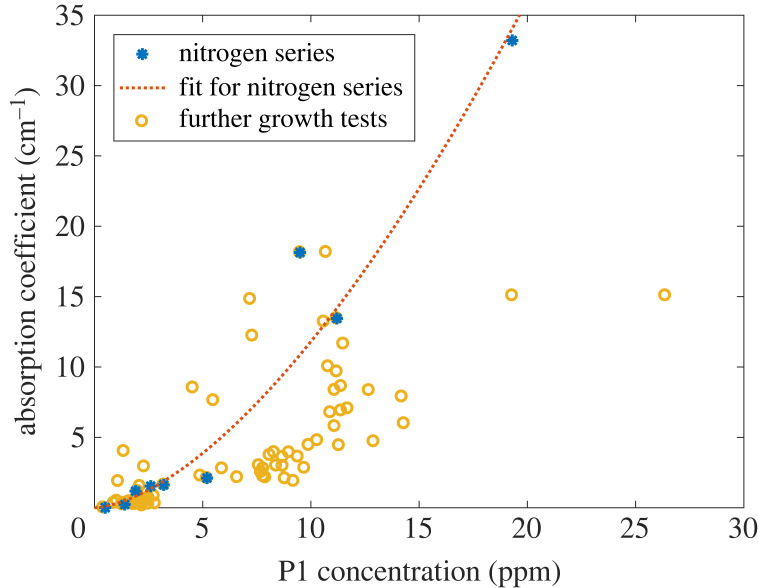
Absorption coefficient (equation (2.1)) for different P1 concentrations in diamond. When growing CVD diamonds with only N/C ratio varying (blue asterisk, ‘Nitrogen series’), their absorption shows a super-linear correlation (orange dashed line) with the concentration of P1 centres. Further growth tests (yellow circle) in the same reactor confirm the positive correlation between absorption and the nitrogen content in diamonds. This correlation can be optimized by locally optimizing the growth parameters for different nitrogen doping levels.

To investigate the link between nitrogen content and diamond absorption, we have grown a nitrogen series by varying the N/C ratio (altered by different values of N${\;}_{2}$ flow for a fixed ${\mathrm{CH}}_{4}$ flow into the plasma) and keeping other conditions fixed. For this series, the absorption coefficient shows a super-linear correlation with the as-grown P1 concentration, figure 1, blue asterisk for the series and orange dashed line for its fit. This clearly indicates that nitrogen doping during the CVD growth attributes to diamond absorption, a higher nitrogen doping level leads to stronger absorption. However, whether the P1 centre is a direct contributor to absorption needs further investigation, which will be discussed in the next section.

This preliminary correlation between P1 centres and absorption can be improved (i.e. to reduce absorption for the same P1 concentration) by optimizing the growth parameters. For that, we have further grown samples in the same reactor with varied individual growth parameters, such as oxygen or methane flow, total gas flow, pressure and holder geometry, which have been discussed in [41,42]. Yellow circles in figure 1 show the result of these growth protocols. By adjusting growth parameters for different P1 concentrations, we can remarkably reduce the absorption compared with the original fit (orange dashed line). Since the original recipe was optimized at a lower nitrogen doping level the potential for improvement at higher nitrogen doping shows that different growth ‘recipes’ are needed for respective P1 concentrations to achieve an optimized crystal quality. Above all, the large number of samples confirms that absorption is positively correlated to the nitrogen content in the diamond, calling for concrete spectral studies for further insight.

### (b) UV–Vis spectral study

**Figure 2.**
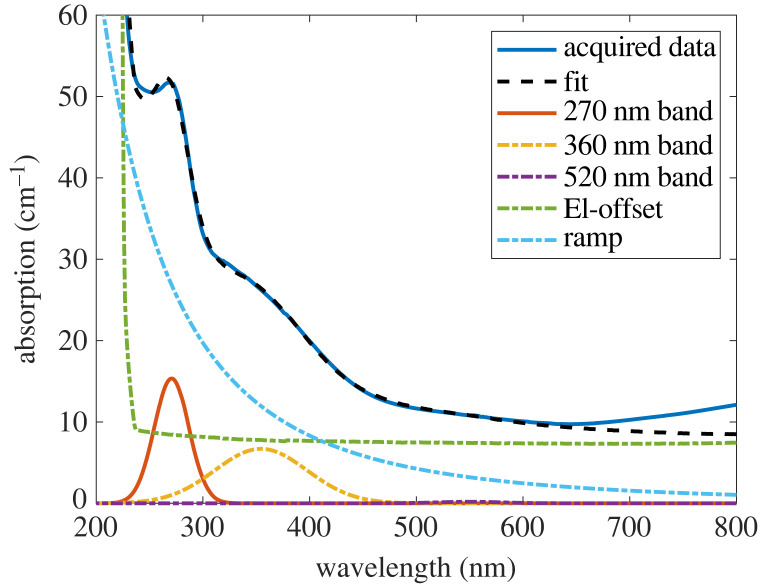
The UV–Vis absorption spectrum and the fit of the nitrogen-doped diamond after growth. The offset in the entire UV–Vis range (‘El-offset’), the ‘ramp’, and a continuous increase starting from 650 nm are the main contributor to the absorption at approximately 700 nm.

To understand the causes of the absorption at 700 nm, we fitted the diamond UV–Vis spectrum and separated it into components to see which are the influencing factors. We used the method introduced earlier in [40] for the fitting (figure 2): the spectrum is separated into five components, including three Gaussian bands (which are centred at 270, 360 and 520 nm), a ‘ramp’ describing the overall decreasing trend of the spectrum and a reference spectrum from a pure diamond (‘El-offset’). Be aware that all samples in figures 1 and 2 are as-grown samples with low NV concentrations, therefore, their NV absorption band (approx. at 400–700 nm) is very weak and has negligible influences.

The 270 nm absorption band links to the P1 centre, which has a small bandwidth that shows no effect on the absorption around 700 nm. This means, although absorption at 700 nm increases with the P1 concentration, the P1 centre is not a direct contributor to the absorption at this regime. The 360 and 520 nm absorption band have been suggested to be related to vacancy clusters and NVH${\;}^{0}$ centres, respectively [43]. Both absorption bands do not reach into the relevant wavelength regime around 700 nm and thus do not contribute to NV signal light absorption.

The spectral fitting indicates three main factors that are accountable for the high absorption: the offset of the spectrum in the entire UV–Vis range (i.e. the fitting coefficient for the ‘El-offset’), the ‘ramp’ and a continuously increasing feature starting from 650 nm. This spectrum is quite representative since these three spectral components are typically the main contributors to the absorption at 700 nm in all samples investigated. The continuously increasing feature starting from 650 nm only appears for the highly nitrogen-doped samples. We now discuss the three features and their potential causes, respectively.

The offset of the entire spectrum is hardly assigned to any single defect but more complex causes link to the diamond crystal quality. One hypothesis points to non-diamond carbon inclusions, which leads to greyish coloration in the diamond and a high absorption in the whole UV–Vis range [33]. Our highly nitrogen-doped samples show a deep grey colour that conforms to this hypothesis, though further verification with Raman spectroscopy is needed. If this assumption is true, the high absorption for increasing nitrogen content is more likely a fundamental issue in the nitrogen-doped CVD growth. Nevertheless, it also means that the appearance of the large spectral offset can be potentially reduced by a high-temperature treatment, especially the HPHT annealing, which can transform carbon inclusions back into diamond.

The ‘ramp’ shows less effect than the offset, it is generally suggested to be vacancy-related [44–46]. The absorption increase in 650–800 nm is significant for samples with high P1 concentrations. This indicates the H2 centre (${\mathrm{NVN}}^{-}$) as a possible candidate: the H2 centre is formed from the P1 centre under similar conditions with the NV centre, meaning a positive correlation between the as-grown concentration of H2 centres and P1 centres can be expected. Since the spectrum was only measured up to 800 nm, whether the H2 centre is the actual cause needs to be further investigated and could be confirmed by extending the absorption spectrum past its ZPL (at 986.3 nm).

From the large number of the growth tests (figure 1), we found that optimizing the growth can reduce the offset of the absorption, and the diamond exhibits less greyish coloration. The ‘ramp’, however, did not show a clear trend with either the nitrogen doping level or other growth parameters. The increasing feature from 650 to 800 nm could be hardly removed by optimizing the growth protocol. This feature seems to always occur for the very high nitrogen doping regime. In this sense, the optimization of absorption in the growth phase should be mainly focused on reducing the offset. Further treatments, e.g. high temperature annealing, can potentially help to reduce the absorption after growth, which will be our future study.

### (c) Influence of irradiation and annealing on absorption

Most of the NV-ensemble-based sensing applications require a high NV concentration, which is hardly achievable in as-grown diamonds. To create more NV centres in the diamond, irradiation and annealing steps are often conducted. Especially electron-beam (e-beam) irradiation is well established for creating single vacancies in bulk diamond plate, a subsequent annealing step then combines vacancies with P1 centres to form NV centres. Although diamond absorption can be optimized during and after growth, the irradiation and annealing treatment are often the last steps that decide the final state of the crystal quality. Therefore, it is necessary to understand their influence on absorption.

**Figure 3.**
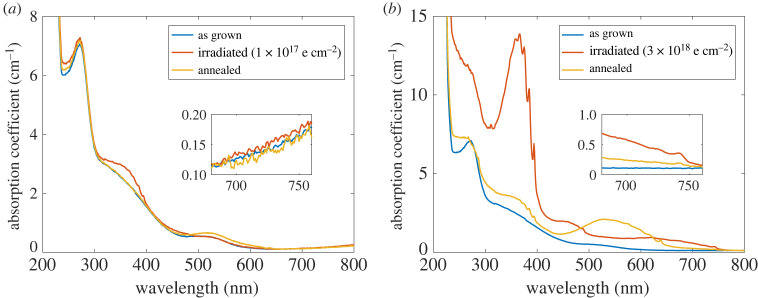
Absorption spectra, respectively, after growth, irradiation and annealing steps for (*a*) $1\times 10^{17}\;e\;{\mathrm{cm}}^{-2}$ and (*b*) $3\times 10^{18}\;e\;{\mathrm{cm}}^{-2}$ irradiation fluence (both with 1 MeV electron energy). The annealing step was conducted with $1000^{\circ }C$ for 2 h.

We here compare two samples irradiated with 1 MeV electron energy, respectively, with $1\times 10^{17}\;e\;{\mathrm{cm}}^{-2}$ (as ‘low fluence’) and $3\times 10^{18}\;e\;{\mathrm{cm}}^{-2}$ (as ‘high fluence’). The two samples have been grown under the same conditions, with an initial P1 concentration of 2.2 ppm, and they were annealed after irradiation simultaneously. We took their UV–Vis spectrum after each step (after growth/irradiation/annealing), figure 3, the absorption coefficient was calculated according to equation (2.2).

For the low irradiation fluence, both the irradiation and annealing show a negligible effect on the absorption at 700 nm, figure 3*a*. The irradiation did not create any defect with an absorption band in this regime, and the newly created NV absorption band after annealing has a ‘tail’ that hardly extends to 700 nm (but ends at around 650 nm). The absorption coefficient of interest stays consistent after the treatment.

By contrast, for the high irradiation fluence, figure 3*b*, the creation of neutral vacancies $V^{0}$ (GR1 centres) significantly increased the absorption at 700 nm, as its absorption band covers a broad range from around 500 to 750 nm. The annealing treatment converted GR1 centres partially into NV${\;}^{0}$ centres, leading to a decrease in absorption. However, GR1 centres were not fully converted, their remaining part still results in a higher absorption than the as-grown phase. Earlier on we have stated that over-irradiation is harmful [46], as it creates more NV${\;}^{0}$ thus deteriorating the NV charge stability and the magnetic resonance contrast. The result here further supports that statement, as over-irradiation remarkably increases the absorption at 700 nm, which results in a higher optical loss in the material. The annealing step can compensate for that to some extent (by converting a part of GR1 centres), only in the sense of pulling down the absorption at 700 nm, but still at the cost of promoting the creation of ${\mathrm{NV}}^{0}$. Consequently, one should avoid over-irradiating the diamond, and an optimized irradiation condition is of great importance to acquire high-quality diamonds with high NV concentrations.

The two samples in figure 3 are representative of multiple CVD samples that we have studied [47]. Nevertheless, the influence of the after-growth treatment is in general much less than the synthesis process. Different nitrogen doping levels can cause orders of magnitude increases in absorption (figure 1), which is way higher than those caused by the GR1 band. The most important role of the treatment is to achieve an improved combination of high NV concentrations and low absorption. An optimized treatment can increase the NV concentration in CVD diamonds by more than an order of magnitude [46], while the absorption at 700 nm remains unchanged. In this sense, the strategy to achieve good combinations of high NV concentration and low absorption is to engineer absorption during the growth, then irradiate the diamond with optimized conditions to enhance the NV concentration.

## 4. Diamond birefringence

### (a) Birefringence for varying nitrogen contents

Diamond is not an inherently birefringent material from its crystal structure. Birefringence is introduced by strain fields that are generated by extended defects and applied stress. It is highly relevant for applications involving cavities, lasers and polarization. Here we look into how nitrogen doping affects birefringence: as doping introduces a different size of the atom, an increased strain could be expected.

**Figure 4.**
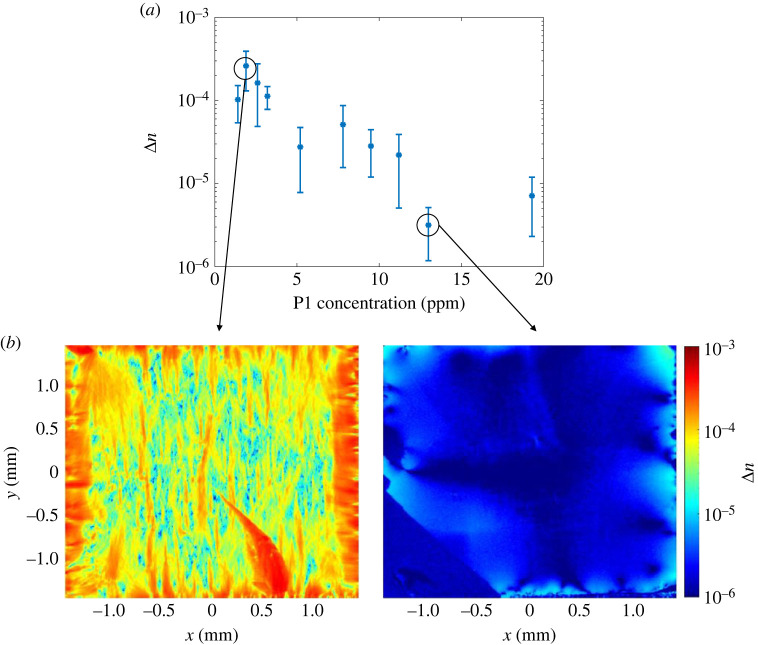
(*a*) Birefringence Δn decreases for increasing P1 concentrations. The error is given by the standard deviation of the birefringence map (the asymmetry is only due to the logarithmic scaling). (*b*) The birefringence map of the sample with highest (left) and lowest (right) average Δn in (*a*), which correspond to the second lowest and the second highest P1 concentration, respectively.

We measured the birefringence Δn of the nitrogen series (as discussed in figure 1) after growth, and plot their average values as a function of the P1 concentration in figure 4*a*. The error of birefringence is given by the standard deviation of the spatial variation in the birefringence map of the respective sample. Both the mean value and standard deviation of the birefringence play roles for the application, as the mean value indicates the total birefringence that an incident beam suffers from, while the standard deviation shows the birefringence inhomogeneity in the sample: an ideal material should, therefore, exhibit a small mean value and a small standard deviation. Contrary to the absorption that shows a positive correlation with the P1 concentration, birefringence shows a remarkable decrease for an increasing P1 concentration. We also observed a higher homogeneity of the birefringence when increasing the P1 concentration, as can be seen in error bars of figure 4*a*. Note that due to the logarithmic scaling on the *y*-axis, a roughly constant size of error bars to the eye with decreasing mean values on the *y*-axis means a significant reduction of the absolute value of the errors. The two samples with the highest P1 concentrations show an average Δn below $10^{-5}$, which has been defined as the standard of ‘ultra low’ birefringence for diamond material in previous work [48]. The theory behind this decreasing trend needs further investigation, but we suggest that either nitrogen atoms can help to prevent the formation of extended defects, or they help to release local stress caused by other defects in the growth. As a consequence, this negative correlation is a positive sign for the aim of creating highly NV-doped diamonds with low birefringence. Figure 4*b* shows, respectively, the birefringence map for a low (left) and high (right) P1 concentration as an example. Both lower values of birefringence Δn and improved homogeneity are clearly visible for the higher nitrogen doping (right), indicating better quality.

Birefringence changes the polarization of a light beam, which can lead to optical losses or reduced precision in set-ups that depend on maintaining a polarization, such as a cavity with elements in Brewster’s angle or polarization-selective excitation of specific NV orientations. We can estimate the worst-case-scenario optical loss by assuming the worst relative orientation between the birefringent axis and polarization direction and assuming that the light shifted out of linear polarization in a single pass is entirely lost. Then the absolute value of the measured birefringence at $\Delta n=10^{-4}$ to 10${\;}^{-5}$ translates to a single-pass light-intensity loss between 0 and a maximum of $\sin^{2}(\pi \;\Delta n\;d\;/\;\lambda )=1.8$–0.018%, respectively, through a diamond thickness of d=300 μm for a wavelength λ=700 nm. This is typically small compared with absorption losses in highly NV-doped diamond [25].

### (b) Influence of irradiation and annealing on birefringence

**Figure 5.**
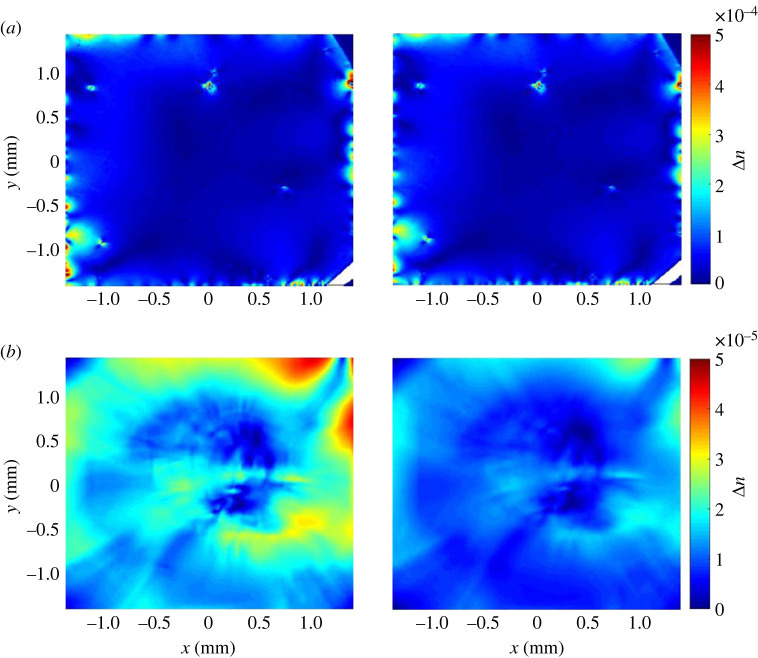
Birefringence before (left) and after (right) irradiation and annealing. (*a*) For thin diamond plates (below 500 μm) no significant change was observed. (*b*) For thick diamond plates (above 1000 μm) a reduction in birefringence over the entire sample area was observed.

We also investigated potential birefringence changes by e-beam irradiation and annealing. The samples were irradiated with 2 MeV electron energy (with different fluences) and then annealed. We compared birefringence before and after treatments in both CVD and HPHT Ib diamonds, figure 5 is representative of the multiple samples that we have investigated [49]. For diamond plates with thickness below 500 μm (both CVD and HPHT diamonds), no significant change in birefringence has been observed before and after the treatment, figure 5*a*. However, in some thick HPHT diamond plates (greater than 1000 μm), reduced birefringence has been observed in the whole sample area, figure 5*b*. The reduction in these thick samples was mainly introduced by the annealing step. It should not originate from the surface changes, but from the changes in the entire depth of the sample, as the thick samples show a more significant effect in the whole area than the thin samples. A possible explanation is that vacancies and their positioning in energetically favourable positions (such as NV centres, etc.) lead to a small release of local stress, which is similar to the assumption that more nitrogen doping releases stress in the crystal (figure 1). To conclude, the irradiation and annealing parameters that we used show a minor and positive (i.e. reducing) influence on diamond birefringence, which benefits the creation of highly NV-doped diamonds with low optical losses.

## 5. Conclusion

In this work, we study diamond absorption and birefringence, with the particular aim to achieve low values for both in diamonds with a high concentration of NV centres. This improves diamond quality for sensing applications in general by reducing optical losses and is essential for diamond lasing, LTM, diamond cavity applications, high-power applications and polarization-dependent applications of NV diamonds.

We found that the absorption at around 700 nm increases remarkably for increasing P1 concentrations; however, the P1 centre itself is not the direct influence factor. The high nitrogen doping level in the growth leads to high absorption in the whole UV–Vis range (as an ‘offset’) and exhibits a continuous increase from 650 to 800 nm, which are the main contributors to the high absorption at the regime of interest. We speculated their causes to be, respectively, non-diamond inclusions and H2 centres (${\mathrm{NVN}}^{-}$), which need further investigation. Optimizing the growth conditions for individual nitrogen concentrations helps to reduce the absorption within the growth process. When treating the diamond for an enhanced NV concentration, one should avoid ‘over-irradiating’ the diamond with too high fluences, as it creates neutral vacancies ($V^{0}$, GR1 band) instead of negatively charged vacancies. These neutral vacancies contribute a large increase of the absorption at 700 nm. This coincides with the requirement for high ${\mathrm{NV}}^{-}/\mathrm{NV}$ ratios, as both the neutral vacancies and neutral NV${\;}^{0}$ state should be avoided, calling for an optimal irradiation fluence as determined in [46]. If this condition is met an increase of absorption can be avoided in the process of irradiation and annealing as no other influences on the absorption have been measured by the two processing steps.

Opposite to the absorption, the average birefringence of the diamond decreases for increasing P1 concentrations, and samples with high P1 concentrations show better homogeneity of the birefringence: we assume that nitrogen atoms help to release the strain during the growth. Electron-beam irradiation and subsequent annealing steps show a minor and if any a positive (i.e. reducing) influence on diamond birefringence.

Overall diamond birefringence is translated to a mostly negligible loss rate, while absorption is a larger factor of optical losses in NV-doped diamond. A compromise between the demand for high P1 densities (and correspondingly high NV densities) and low absorption must be found for cavity applications. The optimization of the absorption and birefringence should be mainly considered during the growth. High-temperature treatments as well as other influences in the growth can potentially reduce the diamond absorption at 700 nm, which will be the direction of our future study.

## Data Availability

This article has no additional data.
